# Evaluating the potential of acupuncture for Alzheimer’s disease treatment: A meta-analysis and systematic review of mouse model studies

**DOI:** 10.1038/s41398-026-03923-9

**Published:** 2026-03-17

**Authors:** Mohan Yang, Liqi Tong, Zhiling Guo, Zhiqun Tan, Todd C. Holmes, Zhaoxia Yu, Xiangmin Xu

**Affiliations:** 1 Department of Statistics, University of California, Irvine, CA 92697 USA; 2 Department of Anatomy and Neurobiology, University of California, Irvine, CA 92697 USA; 3 Susan Samueli Integrative Health Institute, and Department of Medicine, University of California, Irvine, CA 92697 USA; 4 Center for Neural Circuit Mapping, University of California, Irvine, CA 92697 USA; 5 Department of Physiology and Biophysics, University of California, Irvine, CA 92697 USA

**Keywords:** Learning and memory, Diseases

## Abstract

Acupuncture is an ancient practice that was developed within the framework of traditional Chinese medicine. While acupuncture has been recently proposed as a therapy for Alzheimer’s disease (AD), acupuncture effects are not well understood in terms of neural mechanisms. Here, we review and examine the studies that used AD mouse models and analyze the experiments where researchers administered electroacupuncture (EA) to AD mice to assess the potential therapeutic impact of acupuncture on disease pathology and cognitive function in controlled laboratory settings. We analyzed 29 relevant PubMed articles published between January 2014 and July 2025. Our results reveal that EA significantly reduces both amyloid-beta (Aβ) and phosphorylated tau (p-tau) levels and neuroinflammatory biomarkers, including molecular signatures for activated microglia and astrocytes in the brain. EA also enhances cognitive functions. While no study directly compared acupoint strategies, the indirect comparisons in our network analysis suggest that GV20 has potential as a therapeutic target for AD. Our present meta-analysis and review of literature add to the evidence of integrative health practices for acupuncture-based Alzheimer’s disease treatment.

## Background

Alzheimer’s disease (AD) is a progressive neurodegenerative disorder that profoundly impacts both individuals and society. AD is characterized by cognitive decline, memory loss, and significant behavioral changes that impose a substantial burden on patients and their families. Globally, over 55 million people are currently living with dementia, with AD being the most common cause. This number is expected to double in about 20 years, reaching 78 million by 2030 and 139 million by 2050 [1].

Despite extensive research and drug development efforts, the search for effective treatments for AD has been disappointing. There is currently no cure for AD. Existing therapeutic candidates fail to demonstrate efficacy in clinical trials [2]. Acupuncture is an important component of traditional Chinese medicine. Acupuncture has recently gained attention for its potential therapeutic effects beyond pain management and now extends to other diseases in the field of neurology. Acupuncture practitioners insert thin needles through the skin to stimulate precise spatial acupoints in the body to achieve therapeutic effects. Modern variations of acupuncture include electroacupuncture (EA), which delivers low electric currents through acupuncture needles in place. While acupuncture is not well understood in terms of physiological mechanisms, it presents a non-addictive, non-pharmacological option for disease treatment. There is growing interest in its potential application for neurodegenerative diseases, including AD [3]. Emerging evidence suggests that EA may exert multifactorial neuroprotective effects, including modulation of neuroinflammation, neurotrophic signaling, synaptic plasticity, and amyloid and tau-related pathology [4, 5].

It is now timely and significant to conduct a rigorous meta-analysis and systematic review to evaluate the potential of acupuncture for AD treatment. Acupuncture studies in humans are complicated by patient expectations and placebo effects. In comparison, rodent models allow for objective evaluations of outcomes following acupuncture treatment and underlying mechanisms. Human acupuncture points have been translated to corresponding acupuncture points in mice and rats [6, 7]. The acupoint localization in animal models follows internationally accepted anatomical transpositional mapping guidelines, including those issued by the Experimental Acupuncture Science Committee of the China Association of Acupuncture-Moxibustion [8]. Substantial evidence shows that the anatomical structures and physiological functions of mice are similar to those in humans. Acupoints along meridians overlying sensory nerves are distributed over the body, which are similar in mice and humans. Previous studies demonstrated that applying acupuncture at specific acupoints can regulate physiological functions through modulating neural circuits in mice, as it does in humans [9–11]. The cross-species mapping relationships have been validated through functional neuroimaging, electrophysiological, and autonomic readouts, showing that stimulation of rodent acupoints such as GV20 and ST36 activates neural circuits analogous to those engaged by corresponding human acupoints [9, 12, 13]. These findings support the functional equivalence of transpositional mapping across species and strengthen the rationale for applying acupuncture paradigms in mouse models. In this study, we aim to provide a comprehensive evaluation of the existing evidence on the efficacy of acupuncture in modulating AD-related features by focusing on mouse model studies. Beta-amyloid (Aβ) and tau pathologies are major biomarkers of AD [14]. In addition, neuroinflammation has been viewed as a key pathophysiological factor in AD [15]. The available data for mouse models include processing and production of Aβ, as well as abnormalities in post-translational modifications and aggregation of the tau protein. The present study focuses on changes in Aβ, phosphorylated tau (p-tau), neuroinflammatory markers, and cognitive performance observed in APP/PS1, 5xFAD, and 3xTg AD transgenic mice following EA-treatments. While these transgenic mouse models do not exhibit the complete neurodegenerative spectrum seen in humans, they remain the extensively validated experimental systems for dissecting disease mechanisms and testing therapeutic hypotheses under experimental conditions [16–18]. We also note that the therapeutic significance of Aβ modulation in our analysis is not intended for endorsing an amyloid-centric hypothesis, but in using Aβ and p-tau as quantifiable biomarkers that reflect broader pathological cascades. Although senescence-accelerated P8 (SAMP8) mice have been used in AD studies as well as acupuncture treatments, the data from these mice are excluded from the analysis due to their unclear genetic background and significant variability in phenotypes [19, 20].

We analyzed 29 relevant PubMed articles published between January 2014 and July 2025. By systematically combining and analyzing data from these studies, we evaluate the therapeutic potential of EA in AD transgenic mouse models. EA is used more frequently than manual acupuncture for animal models [21]. The key outcome measures of the acupuncture treatments include treatment-induced changes in biomarkers such as Aβ, p-tau, interleukin 1 beta (IL-1β), glial fibrillary acidic protein (GFAP), and ionized Calcium Binding Adapter Molecules 1 (IBA1), as well as cognitive behavior-related metrics such as time spent in the target quadrant, number of platform crossings, escape latency, and discrimination index. Data extraction was facilitated by Webplot Digitizer [22] and a novel web application to ensure the accuracy of measurements from published data figures. Standardized mean differences (SMD) were calculated to assess the effect sizes. SMDs of 0.2, 0.5, and 0.8 are considered small, medium, and large, respectively. We chose SMD because it is the natural choice when different instruments or scales are used across studies. Even when the same scale is used in all studies, SMD is still recommended due to its superior generalizability [23]. A network meta-analysis was performed to compare the relative efficacy of various acupoint combinations.

One challenge in examining the effect of acupuncture on AD arises from the complexity of the disease itself, coupled with the heterogeneity of acupuncture treatments. Our meta-analysis and systematic review focus on investigating the influence of acupuncture on AD phenotypes using transgenic mice, as they offer unique advantages for investigating therapeutic interventions such as EA. Transgenic mouse models replicate many of the key pathological features of AD, such as Aβ accumulation, tau hyperphosphorylation, and neuroinflammation. Studies using these mouse models allow for controlled experimental conditions and precise manipulation of variables that are not feasible in human studies. In addition, animals are much less susceptible to placebo effects, which can significantly confound results in human trials. Our meta-analysis and systematic review provide an unbiased evaluation of the therapeutic potential of acupuncture in AD.

## Methods

### Literature selection process

**Fig. 1:**
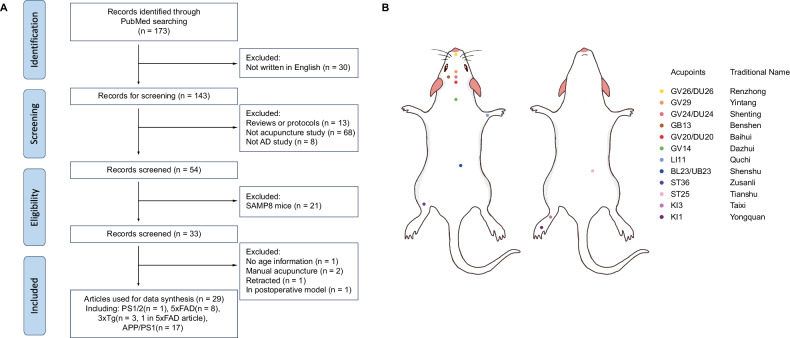
Study selection process and mouse-human acupoint correspondence. **A** The workflow diagram to show how articles were filtered based on specific criteria, including study type, animal model, and language. **B** The spatial positions of animal acupoints in the mouse model, which corresponds to the analogous anatomic sites of human acupoints.

PubMed articles from January 5, 2014, until July 2025, were searched with the following combined terms (Alzheimer’s AND disease) AND (acupuncture OR electroacupuncture) AND (transgenic mice OR animal model OR amyloid mice OR APP mice OR APP/PS1 mice OR 3xTg mice OR 5xFAD mice OR 5 Familial mice OR Tau mice) NOT (rat). In total, 172 results were found. As shown in Fig. 1A, we filtered out articles based on the following exclusion criteria: reviews or protocols, no acupuncture treatment, non-AD study, SAMP8 mice only, no age information, retracted, or in the postoperative model. There has been a predominant focus on the APP/PS1 and SAMP8 models, with fewer studies involving the 3xTg and 5xFAD models. We excluded SAMP8 mice due to their distinct aging characteristics, which may not accurately resemble the genetic influence on AD pathology. SAMP8 models were excluded to ensure model homogeneity and experimental reproducibility across datasets used in quantitative synthesis. In addition, we focused on the studies published in English to ensure methodological accuracy and reliable quantitative data extraction. While this may introduce language bias, the non-English reports were excluded because key experimental details (e.g., stimulation parameters, cognitive tests, statistical variance) could not be consistently verified across translations. After excluding articles with the criteria described above, 29 papers were ultimately included in our meta-analysis (Supplementary Table 1): ([4, 24–27]).

The spatial positions of animal acupoint treatments used in the included papers are illustrated in Fig. 1B. As established in earlier studies for acupuncture research modeling, a transpositional method is used to locate animal acupoints on the surface of animal skin corresponding to the analogous anatomic sites of human acupoints [6]. Histological investigations have shown that acupoints have a higher concentration of neural and vascular elements, including nerve endings, A and C-afferent fibers, and microvessels, than their surrounding locations [7, 28]. Thus, the acupoints may be more sensitive to stimulation, such as the mechanical stimulation of the needle and the electrical current stimulation of electroacupuncture.

### Outcome measurements

In this meta-analysis, we analyzed commonly measured biomarkers and behavioral outcomes. To visualize the distribution of outcome variables across the selected papers, two Venn Diagrams were generated: one for biomarkers and one for behavioral outcomes. The biomarker Venn Diagram (Supplementary Figure S1 A) focuses on Aβ deposition, p-tau, inflammatory cytokine IL-1β, IBA1, and GFAP. The behavioral Venn Diagram (Supplementary Figure S1 B) focuses on four behavioral metrics (time spent in the target quadrant, number of platform crossings, escape latency, and discrimination index). Each number within the diagram represents the count of the studies that contain the specific combination of these outcome variables.

### Data extraction

For each included paper, we recorded data on each estimate, the sample sizes, the experimental conditions (e.g., acupoints or their combinations), animal characteristics (age and transgenic model), and EA intensity and frequency. Most estimates are presented in bar plots with standard deviations. We used Webplot Digitizer [22] and our in-house web application AutoExtract, a user-friendly web application available at GitHub (Link: https://github.com/HenryYang03/AutoExtract) to extract data from bar plots. AutoExtract was developed by training a YOLOv5 model for bar/axis detection based on over 200 annotated figures and then using PyTesseract for optical character recognition to locate the key information to be used to compute the mean and standard deviations.

### Statistical methods

The data from 29 research papers were systematically extracted, with each biomarker or behavioral outcome data file following a specific naming convention. Each study was reviewed to evaluate the accuracy of reported values and consistency in the definitions of control and treatment groups. Any discrepancies or inconsistencies in the data were resolved through thorough manual cross-checking. This process determined whether the extracted data were suitable for meta-analysis.

We first aggregated the common biomarkers or behavioral outcomes, as described in the outcome measurement section, across all studies into distinct datasets. We then split these datasets between the control group (transgenic AD model) and the treatment group (transgenic AD model + EA). To accurately calculate the standardized mean difference (SMD), we employed the inner join method to ensure precise pairing of the treatment and control groups based on covariates such as age, sex, and acupoints. Each effect size was then calculated as the SMD between the treatment and control groups, along with the standard error of the SMD, to measure both the magnitude of the effect and variability.

Meta-analyses were performed using the R package ‘metafor’ [29]. When estimating the overall effect of EA across studies, we apply a random-effects model. Random-effect models account for between-study variability and potential heterogeneity. This is expected due to the inherent differences in study designs, populations, and experimental conditions across the studies. Key metrics such as heterogeneity indices (e.g., *I*^2^ and *P value*) were computed to evaluate the degree of variability and the robustness of the results. Potential biases, including publication bias, were assessed using funnel plots and Egger’s test to ensure the reliability of the conclusions drawn.

Direct comparisons between different EA strategies were not feasible as each study examined only one specific combination of acupoints. We employed network meta-analysis using the R package ‘netmeta’ [30] to address this limitation. This approach allows for indirect comparisons between different combinations of EA at different acupoints and evaluation of the relative efficacy of various acupoint strategies. The network meta-analysis provided a comprehensive framework for assessing the hierarchy of interventions and extended the scope of pairwise comparisons.

## Results

In this section, we first provide meta-analysis results from screening the published studies. We then assess and address the potential caveat of publication bias. Finally, in addition to examining the effectiveness of EA, we present a network meta-analysis to understand whether the effects of EA vary across different acupoint combinations.

### Meta-analysis results

We analyzed the influence and effects of EA on AD phenotypes using transgenic mouse models of AD. To assess the potential therapeutic effects of EA, we examine several key AD biomarkers and behavioral metrics associated with AD. Aβ, particularly Aβ42, is one of the key components of Aβ plaques, a hallmark of AD pathology. Phosphorylated tau (p-tau), a marker of tau hyperphosphorylation and neurofibrillary tangle, represents another core pathological feature of AD. IL-1β, a pro-inflammatory cytokine, has been shown to associate with neuroinflammation along with activated microglia. GFAP (a marker of astrocyte activation) and IBA1 (a marker for microglia activation) are elevated in AD and contribute to neurodegeneration. In addition to biomarkers, we also examine three behavioral metrics related to learning and memory, including time spent in the target quadrant, number of platform crossings, escape latency, and discrimination index. These assessments are critical for determining how EA might alleviate cognitive deficits associated with AD.

As detailed below, our results reveal that acupuncture significantly reduces amyloid-beta (Aβ) levels [standardized mean difference, SMD = −1.13; 95% CI (−1.38, −0.88)] and neuroinflammatory biomarkers, including IBA1 [SMD = −1.38; 95% CI (−2.08, −0.67)) in the brain. EA enhances cognitive functions, as evidenced by increased time spent in the target quadrant [SMD = 1.20; 95% CI (0.79, 1.60)] and other measurements. Please note that standardized mean differences (SMD)s of 0.5–0.8 and >0.8 are often considered medium and large effect sizes, respectively.

#### Aβ deposition in all brain regions

**Fig. 2:**
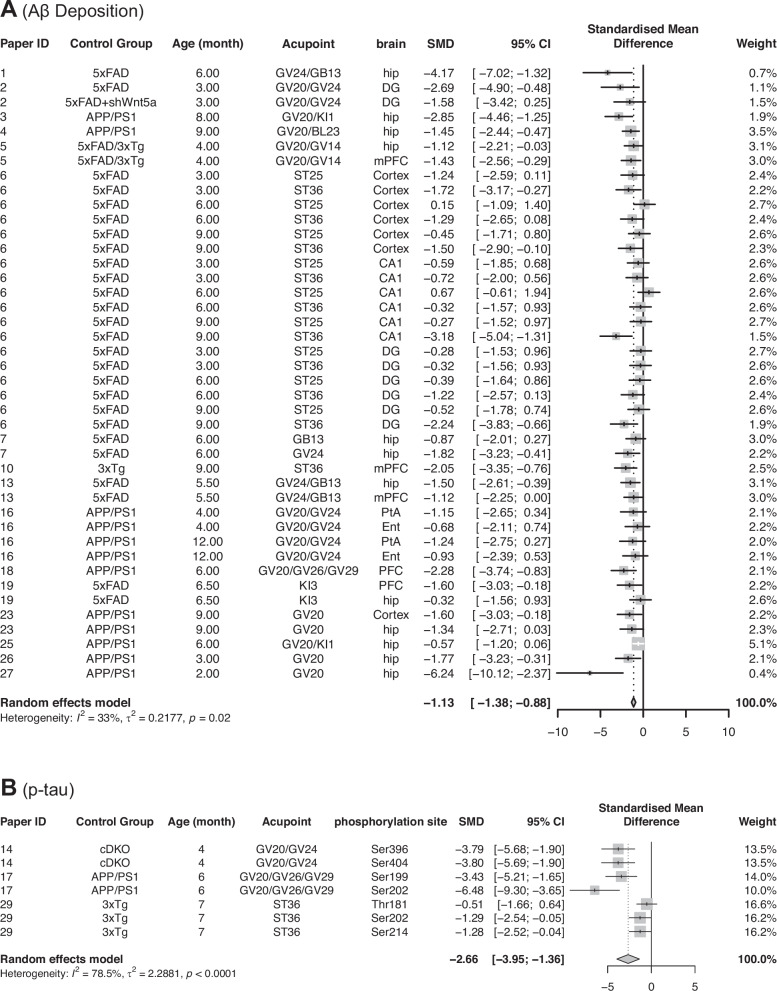
Meta-analysis of electroacupuncture effects on amyloid-β deposition and phosphorylated tau. **A** Forest plot for the effect of EA treatment on Aβ deposition. **B** Forest plot for the effect of EA treatment on p-tau. (SMD = (Treatment Group’s outcome - Control Group’s Outcome)/SD pooled).

The effects of EA on Aβ deposition levels were reported in 16 articles. Based on the experimental conditions [age (3–12 months old), acupoints (ST25, ST36, GV24, GB13, and more), and brain region (hippocampal formation and prefrontal cortex)], 42 comparisons were made, where each compared the Aβ deposition level of the treatment group with the control group under the same experimental conditions. The meta-analysis results reveal that the Aβ deposition level is significantly lower in the treatment group (5xFAD+EA, 5xFAD+shWnt5a+EA, 3xTg+EA, APP/PS1 + EA, 5xFAD/3xTg+EA) compared to the control group (5xFAD, 3xTg, 5xFAD+shWnt5a, APP/PS1, 5xFAD/3xTg). [SMD = −1.13; 95%CI (−1.38, −0.88); Z = −8.77; *P value* < 0.0001; I^2^ = 33%] (Fig. 2A). The significant reduction in Aβ deposition suggests that EA could have a strong effect in reducing one of the primary pathological markers of Alzheimer’s disease.

#### Aβ deposition in distinct brain regions

To explore potential differences in Aβ deposition across distinct brain regions, a subgroup analysis was conducted. The brain areas were categorized into four subgroups: General Hippocampus (hip, CA1, DG), Prefrontal Cortex (PFC, mPFC), General Cortex (Cortex, PtA), and Entorhinal Cortex (Ent) (Table 1). The forest plot for each subgroup is provided in Supplementary Figure S2A–D.

**Table 1:** Subgroup analysis of EA effects on Aβ deposition.

Subgroups	Number of comparison	SMD	95%CI for SMD	I^2^
General Hippocampus	k = 26	−1.0963	[−1.458, −0.734]	47.8%
Prefrontal Cortex	k = 5	−1.6279	[−2.194, −1.062]	0%
General Cortex	k = 9	−1.0547	[−1.515, −0.595]	0%
Entorhinal Cortex	k = 2	−0.8013	[−1.821, 0.218]	0%

Notably, the effect of EA on Aβ deposition appears to be the greatest in the prefrontal cortex. While the Entorhinal Cortex (k = 2, SMD = −0.8013, 95% CI: −1.8207 to 0.2182, I² = 0%) includes confidence intervals that contain zero, we note that only two comparisons were available for meta-analysis. Nevertheless, these differences may imply potential variability of treatment response across different brain regions. This suggests the need for further studies.

#### Tau pathology

To evaluate the effect of EA on Tau pathology, we pooled four estimates of phosphorylated tau (p-tau) levels across two studies. Figure 2B shows that p-tau level is significantly lower in the treatment group (cDKO+EA, APP/PS1 + EA, 3xTg+EA) compared to the control group (cDKO, APP/PS1, 3xTg). [SMD = −2.66; 95%CI (−3.95, −1.35); Z = −4.02; *P value* < 0.0001; I² = 78.5%]. Between-study heterogeneity was high (I² = 78.5%). Inspection of the study-level effect indicates that this heterogeneity was driven by the most recent paper [27], which reports smaller reductions in p-tau (SMDs = −0.51 to −1.29 across Thr181/Ser202/Ser214) than the earlier studies [31, 32]. It should be noted that these findings are derived from only three studies and were obtained in non-tau transgenic mouse models. Further research is required to validate the effect of EA on tau pathology.

#### Glia activities

**Fig. 3:**
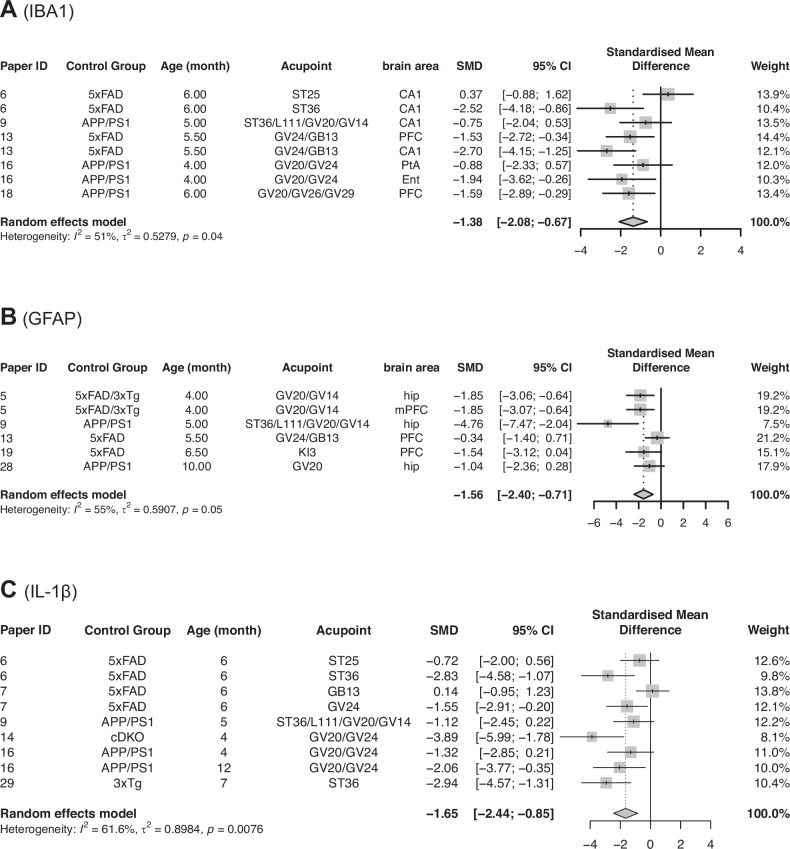
Meta-analysis of electroacupuncture effects on neuroinflammatory markers (IBA1, GFAP, and IL-1β). **A** The forest plot for the effect of EA on microglia activation, IBA1 level. **B** The forest plot for the effect of EA on GFAP levels. **C** The forest plot for the effect of EA on IL-1β. (SMD = (Treatment Group’s outcome – Control Group’s Outcome)/SD pooled).

Microglia are activated in response to brain injury. For microglia activation, we combined eight estimates of IBA1 levels from five studies. Figure 3A shows that the IBA1 levels are significantly lower in the treatment groups (5xFAD+EA, APP/PS1 + EA) compared to the control groups (5xFAD, APP/PS1) [SMD = −1.38; 95% CI (−2.08, −0.67); Z = −3.83; *P value* = 0.0001; I² = 51%].

The other glia marker we examined is GFAP, an intermediate filament marker for astrocytes. In five papers that measured GFAP protein, six estimates were reported. The combined estimate shows that GFAP level is significantly lower in the treatment group (5xFAD+EA, APP/PS1 + EA, 5xFAD/3xTg+EA) compared to the control group (5xFAD, APP/PS1, 5xFAD/3xTg). [SMD = −1.56; 95%CI (−2.40, −0.71); Z = −3.6; *P value* = 0.0003; I^2^ = 55%]. (Fig. 3B).

Since elevated glia activity is often linked to chronic inflammation and neurodegeneration, the observed decrease in IBA1 and GFAP levels suggests that EA may be effective in reducing neuroinflammatory processes, thereby contributing to the overall neuroprotection and alleviation of AD pathology.

#### Inflammatory biomarkers: IL-1β

Our meta-analysis examined the effects of EA on IL-1β, a pro-inflammatory biomarker. The estimate for IL-1β, based on nine estimates from six papers, indicates that IL-1β level is significantly lower in the treatment group (5xFAD+EA, APP/PS1 + EA, cDKO+EA, 3xTg+EA) compared to the control group (5xFAD, APP/PS1, cDKO, 3xTg). [SMD = −1.65; 95%CI (−2.44, −0.85); Z = −4.04; *P value* = <0.0001; I^2^ = 61.6%] (Fig. 3C).

#### Behavioral analysis: Number of platform crossed as a measurement of spatial learning and memory

**Fig. 4:**
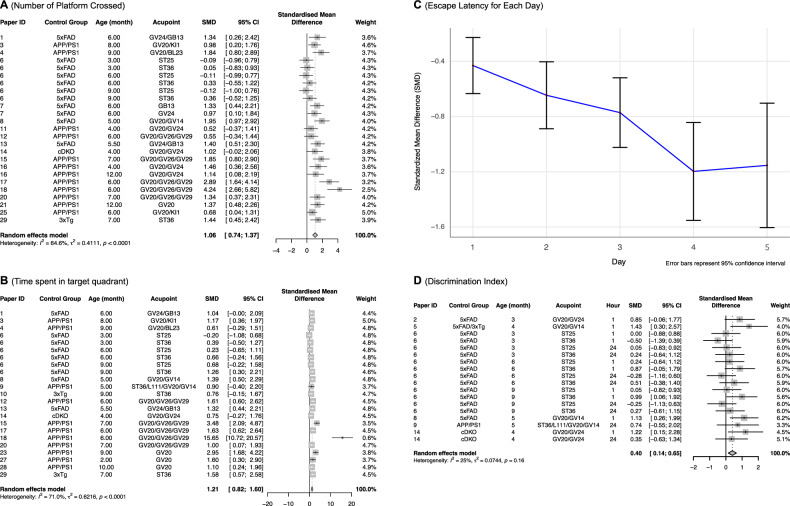
Meta-analysis of electroacupuncture effects on cognitive and memory performance in behavioral tests. **A** The forest plot of the EA treatment effect on the number of platform crossings. **B** The forest plot of the EA treatment effect on the time spent in the target quadrant. **C** The random effects model’s SMD result for escape latency across multiple days, demonstrating a faster learning curve for mice receiving EA. **D** The forest plot for the effect of EA treatment on the discrimination index. The hour column represents the amount of time that the mice were put back into the same experiment box after the training. (SMD = (Treatment Group’s outcome – Control Group’s Outcome)/SD pooled).

Among the 18 papers that analyzed the number of platforms crossed, 25 comparisons were reported between the EA and control groups. The meta-analysis result is shown in Fig. 4A. Although the estimates from one article (paper ID 6) contained outlier data that is inconsistent with the other papers, the overall estimate by combining all comparisons indicates that the number of platforms crossed is significantly higher in the EA group (5xFAD+EA, APP/PS1 + EA, cDKO+EA, 3xTg+EA) compared to the control group (5xFAD, APP/PS1, cDKO, 3xTg). [SMD = 1.06; 95% CI (0.74, 1.37); Z = 6.56; *P value* < 0.0001; I^2^ = 64.6%]. The observed increase in the number of platforms crossed suggests that EA may enhance spatial learning and memory in AD transgenic mice. As a result, EA might have a positive effect on the cognitive functions that are typically impaired in AD.

#### Behavioral analysis: Time spent in target quadrants - a measurement of spatial learning and memory

Among the 18 papers that recorded time spent in the target quadrant, 23 estimates were reported for the effects of EA. Most of the estimates indicated EA increased time spent in the target quadrant. The combined result through meta-analysis, shown in Fig. 4B, reveals that time spent in the target quadrant is significantly higher in the EA treatment group (5xFAD+EA, APP/PS1 + EA, 3xTg+EA, cDKO+EA) compared to the control group (5xFAD, APP/PS1, 3xTg, cDKO). [SMD = 1.21; 95% CI (0.82, 1.60); Z = 6.07; *P value* < 0.0001; I^2^ = 71%]. The increased time spent in the target quadrant suggests that EA may improve memory retention in AD transgenic mice, as this metric is often associated with better spatial memory performance.

#### Behavioral analysis: Escape latency as a measurement of spatial learning and memory

We extracted 30 estimates from 24 papers that reported an effect of EA on escape latency. On Day 1, the escape latency is slightly lower in the treatment group (5xFAD+EA, APP/PS1 + EA, 3xTg+EA, cDKO+EA) compared to the control group (5xFAD, APP/PS1, 3xTg, cDKO). [SMD = −0.47; 95% CI (−0.68, −0.26); Z = −4.4; *P value* < 0.0001; I^2^ = 36.4%]; the decrease continues until Day 4 [SMD = −1.20; 95% CI (−1.55, −0.86); Z = −6.85; *P value* < 0.0001; I^2^ = 72.5%] (Supplementary Figure S3A–E). A line plot with 95% CI for the SMD from Day 1 to Day 5 is created using meta-analysis results (Fig. 4C). This plot indicates that the escape latency of the treatment group decreases at a faster rate compared to the control group over days. The reduction in escape latency implies that EA may accelerate learning and memory acquisition in AD transgenic mice.

#### Behavioral analysis: Discrimination Index as a measurement of spatial learning and memory

18 estimates of the treatment effect of EA on the discrimination index were reported across 6 papers. Our meta-analysis finds that the discrimination index might be higher in the treatment group (5xFAD+EA, APP/PS1 + EA, 5xFAD/3xTg+EA, cDKO+EA) compared to the control group (5xFAD, APP/PS1, 5xFAD/3xTg, cDKO). [SMD = 0.40; 95% CI (0.14, 0.65); Z = 3.08; *P value* = 0.0021; I^2^ = 25%] (Fig. 4D). The combined estimate indicates that EA may have a significant effect on improving mice’s ability to distinguish between familiar and novel stimuli.

### Publication bias

One major concern in a meta-analysis is publication bias, which occurs when studies with significant or favorable results are more likely to be published than studies with non-significant or unfavorable outcomes, aka “negative results”. We performed Egger’s regression test to assess potential publication bias for each outcome. The test indicates potential asymmetry in the funnel plots for Aβ deposition, IL-1β, time spent in the target quadrant, escape latency on Day 4, discrimination index, and number of platforms crossed, which suggests that the meta-analyses may be affected by publication bias (Supplementary Table 2).

We then adopted the trim-and-fill approach to correct publication bias. The effects of EA on Aβ deposition, number of platforms crossed, time spent in the target quadrant, escape latency, and discrimination index remain significant, but the confidence interval for IL-1β includes zero (Table 2), suggesting that this result may no longer be statistically significant. This implies that the original findings, particularly IL-1β, could be influenced by publication bias.

**Table 2:** Trim and fill correction of publication bias.

Previous Aβ Deposition	After Trim and Fill
95%CI for SMD: (−1.38, −0.88)	(−1.12, −0.56)
**Previous Number Platform Crossed**	**After Trim and Fill**
95%CI for SMD: (0.74, 1.37)	(0.42, 1.13)
**Previous Time Spent in Target Quadrant**	**After Trim and Fill**
95%CI for SMD: (0.82, 1.60)	(0.40, 1.30)
**Previous Escape Latency Day 4**	**After Trim and Fill**
95%CI for SMD: (−1.55, −0.86)	(−1.08, −0.28)
**Previous Discrimination Index**	**After Trim and Fill**
95%CI for SMD: (0.14, 0.64)	(0.05, 0.58)
**Previous IL-1β**	**After Trim and Fill**
95%CI for SMD: (−2.44, −0.85)	(−1.73, 0.04)

### Sensitivity and heterogeneity analysis

Outlier removal was performed to evaluate the robustness of the pooled effect. Using the outlier removal method, studies were identified as outliers if their 95% confidence intervals did not overlap with the CI of the pooled effect. Specifically, a study was considered an outlier if the upper bound of its CI was lower than the lower bound of the pooled effect CI, indicating an extremely small effect size, or if the lower bound of its CI was higher than the upper bound of the pooled effect CI, indicating an extremely large effect size. This approach helps to detect studies with effect sizes that differ significantly from the overall effect, which could potentially bias the meta-analysis results. (Supplementary Figure S4A–H) After performing the sensitivity analyses, the pooled effect estimates remained stable and were not largely influenced by outliers.

Among the meta-analysis results, the outcomes for the number of platforms crossed (I^2^ = 64.6%, τ^2^ = 0.41) and time spent in the target quadrant (I^2^ = 71%, τ^2^ = 0.62) exhibited substantial heterogeneity. The corresponding τ^2^ values and significant Q-test results confirmed notable between-study variability. Therefore, subgroup analyses were performed for these two outcomes.

For the number of platforms crossed meta-analysis, the included acupoints were categorized into two groups: the ST group and the non-ST group. (Supplementary Figure S5A, S5B) The non-ST group showed a significantly greater effect (k = 18, SMD = 1.37, 95% CI: 1.04 to 1.69, I² = 50.5%) compared with the ST group (k = 7, SMD = 0.24, 95% CI: −0.15 to 0.62, I² = 23%). Heterogeneity within both subgroups was markedly reduced, and the test for subgroup differences was significant (Q = 19.39, *P value* < 0.0001).

We further examined the heterogeneity within the non-ST group by subgrouping studies according to the animal model, 5xFAD vs. non-5xFAD. (Supplementary Figure S5C, S5D) Results showed that additional subgrouping by animal model did not reduce heterogeneity, and the test for subgroup differences was not significant (Q = 0.00, *P value* = 0.969). Subgrouping by age (<6 months vs. >=6 months) yielded similar results to the animal model subgroup analysis, with no meaningful reduction in heterogeneity.

Leave-one-out influence analysis for time spent in the target quadrant (Supplementary Figure S6A) flagged a single influential study (study 18). Removing study 18 reduces the heterogeneity from I^2^ = 72% to I^2^ = 50% and yielded the meta-analysis result: [SMD = 1.1; 95%CI (0.8, 1.39); Z = 7.3; *P value* < 0.0001; I^2^ = 50%] (Supplementary Figure S6B). Subgrouping by acupoint category again reduces within-group heterogeneity and shows a significant between-group difference (Q = 8.47, *P value* = 0.0036). (Supplementary Figure S6C, S6D). The non-ST group shows a significantly greater effect (k = 13, SMD = 1.4, 95% CI: 1.03 to 1.77, I² = 43.5%) compared with the ST group (k = 9, SMD = 0.65, 95% CI: 0.30 to 1.00, I² = 18.5%).

Across both outcomes, acupoint category (ST vs non-ST) accounts for a substantial share of the variability, and for time in the target quadrant, an additional contribution arises from a single influential study. It is noteworthy that most of the ST group’s results were derived from a single study, and the 95% CI for the number of platforms crossed in the ST group contains 0, suggesting that the ST acupoint treatment might not be effective for the number of platforms crossed.

### Network meta-analysis

Based on the empirical practice of traditional Chinese Medicine, the combination of acupoints may strengthen the therapeutic effects of acupuncture. Therefore, in addition to examining the effectiveness of EA, it is interesting to examine whether the effects of EA vary across different acupoint combinations. Since each study assessed one or a few acupoint combinations, we used network meta-analysis to indirectly compare different acupoint combinations. The control group, which does not receive acupoint stimulation and has an SMD of 0, serves as the baseline to assess the therapeutic impact of different EA combinations on AD transgenic mice. This approach allows us to make direct and indirect comparisons to identify the most effective EA interventions using AD transgenic mouse models.

#### Aβ deposition

**Fig. 5:**
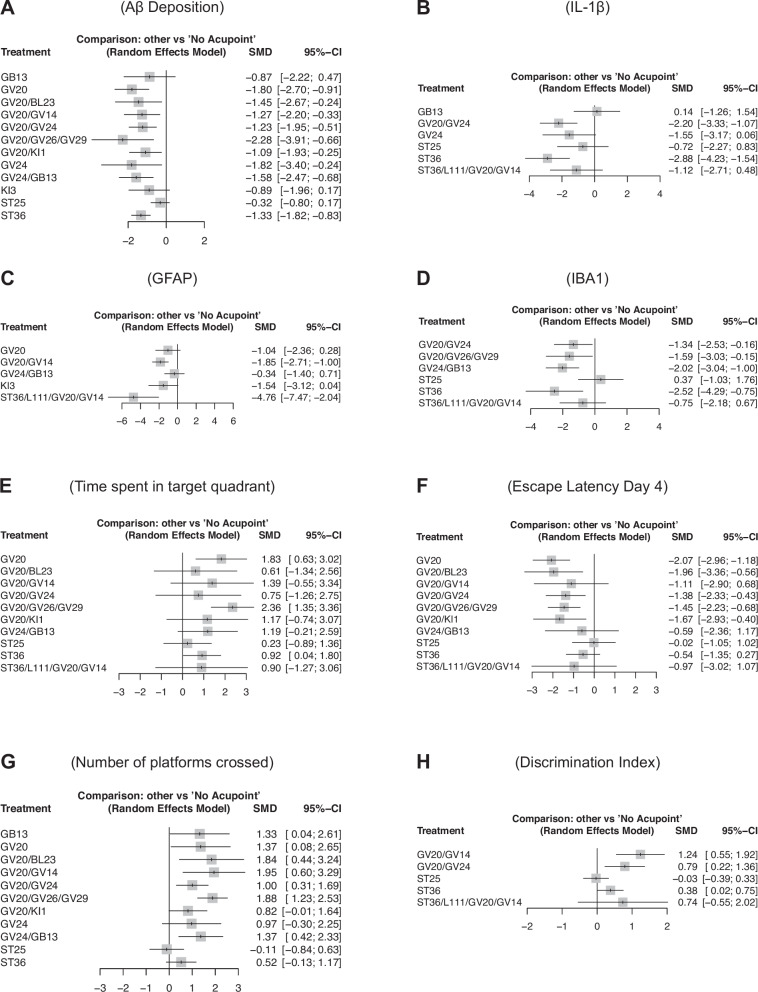
The network meta-analysis compares different acupoint combinations to identify optimal treatments for cognitive and biomarker improvements. **A**–**D** Forest plots that highlight the direct effects of various acupoints on Aβ deposition, IL-1β, GFAP, and Microglia levels, respectively. **E**–**H** Forest plots of the effect of EA at different acupoints on time spent in the target quadrant, escape latency, number of platform crossings, and discrimination index.

The forest plot in Fig. 5A reveals that the EA combination at GV20/GV26/GV29 acupoints exhibits the most substantial reduction in Aβ levels relative to the control group, with an SMD of −2.28 [95% CI (−3.91, −0.66)]. This finding suggests that EA combination at these multi-acupoints holds significant potential in mitigating amyloid pathology, a hallmark feature of AD. In contrast, EA at a single-acupoint GV20 demonstrates a weaker, albeit still significant effect [SMD = −1.80; 95% CI (−2.70, −0.91)], indicating that while single-point interventions can be effective, multi-acupoint stimulation may offer enhanced therapeutic benefits. EA at acupoints such as ST25 [SMD = −0.32; 95% CI (−0.80, 0.17)], GB13 [SMD = −0.87; 95% CI (−2.22, 0.47)], and KI3 [SMD = −0.89; 95% CI (−1.96, −0.17)] shows less pronounced effects, with confidence intervals overlapping 0, suggesting uncertain or weaker efficacy.

#### Neuropathological markers: Inflammatory cytokines, astrocyte activation, and microglia activation

For inflammatory cytokine IL-1β, astrocyte activation (GFAP), and microglia activation (IBA1), the EA combination at ST36/L111/GV20/GV14 demonstrates the strongest treatment effect for IL-1β, while EA at ST36 alone shows the highest efficacy for glia activation (Fig. 5B–D). These findings suggest the potential of EA at ST36 in targeting inflammation-related biomarkers and microglial activation, which are critical in AD pathology.

#### Behavioral outcomes

Memory retention, assessed by the time spent in the target quadrant (Fig. 5E), demonstrates significant improvements with EA combinations at certain acupoints. EA at GV20/GV26/GV29 shows the strongest effect [SMD = 2.36; 95% CI (1.35, 3.36)], indicating its robust impact on enhancing spatial memory. Similarly, EA at the single-acupoint GV20 exhibits a notable increase [SMD = 1.83; 95% CI (0.61, 3.02)], although it is weaker than the combined EA at GV20/GV26/GV29. In contrast, single or combined EA at other acupoints, including GV20/BL23, GV20/GV14, GV20/GV24, GV20/KI1, GV24/GB13, ST25, ST36, and ST36/LI11/GV20/GV14, has confidence intervals overlapping 0, suggesting inconsistent or weaker cognitive benefits.

Escape latency (a measure of memory acquisition) (Fig. 5F) shows that EA at GV20 alone produces the largest reduction [SMD = −2.07; 95% CI (−2.96, −1.18)] with a narrow confidence interval. This suggests a reliable therapeutic impact of single-point EA for this parameter. EA combinations involving GV20 and other acupoints yield weaker effects, reflecting the possibility of diminished efficacy when additional points are combined with GV20. EA at acupoint ST25 or ST36 alone displays the weakest effects (SMD = −0.02 and −0.54, respectively).

The number of platforms crossed is an indicator of spatial learning and memory. As shown in Fig. 5G, the combined EA at GV20/GV14 produces the highest effect [SMD = 1.95; 95% CI (0.60, 3.29)], suggesting that EA at these acupoints together may provide synergistic benefits. The EA combinations at GV20/GV26/GV29 also show a strong positive effect [SMD = 1.88; 95% CI (1.23, 2.53)] with a much narrower CI, suggesting its potential as a key intervention for cognitive enhancement. Conversely, EA at ST25 or ST36 alone yields weaker effects (SMD = −0.11 and 0.52, respectively).

For discrimination index outcomes, the EA combination at GV20/GV14 again shows the largest effect [SMD = 1.24; 95% CI (0.55, 1.92)] (Fig. 5H), while EA at ST25 or ST36 alone remains less effective with SMDs of −0.03 and 0.38, respectively. Overall, behavioral outcomes indicate that EA at GV20, GV20/GV26/GV29, and GV20/GV14 are among the most effective, whereas EA at ST25 or ST36 alone consistently shows weaker effects.

#### Network plot summary

**Fig. 6:**
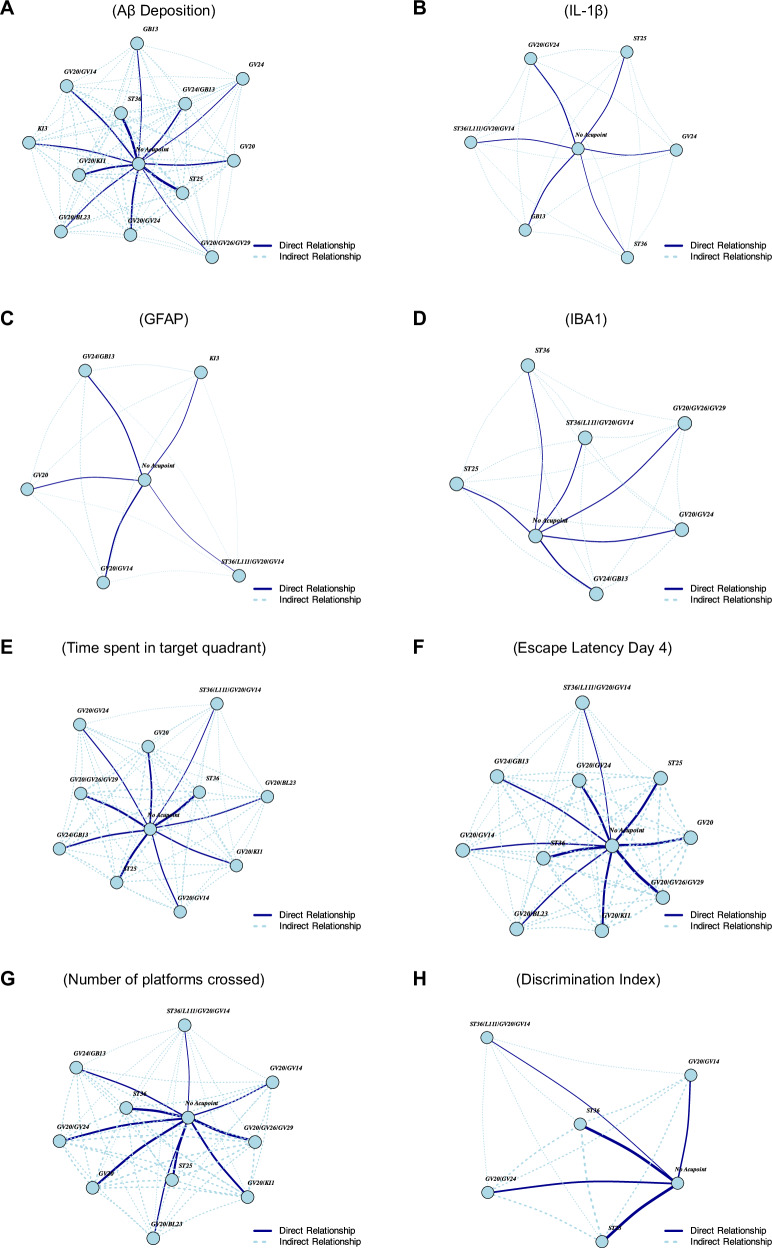
The network plots provide a visual summary of the relationships between acupoints and the outcomes of their combinations. In these plots, the edge widths represent the inverse of the standard error (1/seTE), indicating the precision of estimates. **A**–**D** Aβ deposition, IL-1B, GFAP, and IBA1 levels. **E**–**H** Behavioral outcomes, including target quadrant time, escape latency, platform crossings, and discrimination Index.

The network plots provide a visual summary of the relationships between acupoints and their therapeutic outcomes across various studies (Fig. 6). Each node represents an individual acupoint or a combination of acupoints used in the interventions, with the “No Acupoint” node serving as the baseline for all direct comparisons. This baseline node (control group) represents the absence of acupoint stimulation and has an SMD of 0, allowing for standardized comparisons across the network.

The edges connecting the nodes represent direct comparisons derived from the studies that explicitly compared the effects of an EA intervention with the “No Acupoint” node. Indirect relationships, depicted by dotted lines, are inferred through the network meta-analysis to enable comparisons between acupoints that were not directly compared in the original studies.

The thickness of each edge is calculated as the inverse of the standard error of treatment effect (1/seTE); thus, edges associated with higher precision (smaller standard error) appear thicker, while edges with lower precision (larger standard error) are thinner. For example, the network plot for escape latency on Day 4 (Fig. 6F) highlights the robust direct effects of EA at GV20/GV26/GV29, as shown by its thick connections to the “No Acupoint” node. This reflects precise and reliable effect measurements in reducing escape latency. In contrast, EA at GV24/GB13 demonstrates a less precise effect estimation, as indicated by the thinner edge connecting it to the baseline. Such thin edges suggest greater variability or uncertainty in the effect estimates, emphasizing the need for cautious interpretation.

## Discussion

In this study, we present our meta-analysis results to evaluate the impact of EA on AD phenotype measurements in mouse models possessing AD-related gene mutations. The heterogeneity of different experiment settings often complicates the precise assessment of the effectiveness of electroacupuncture on AD. We tackled heterogeneity in both the selection of published studies and in the analysis method (by employing random-effects models and subgroup analysis). Unlike the existing meta-analysis by Wu et al. 2023 [5] which combined studies from heterogeneous settings and used a similar number of publications as we did, our analysis specifically focuses on transgenic mouse models. Compared to Wu et al.’s study, which only yields the conclusion that EA enhances pro-inflammatory biomarkers, our findings revealed that, in addition to the pro-inflammatory biomarkers, EA has a multifaceted effect on the biomarkers of AD, including Aβ deposition and p-tau, and improved cognitive functions due to EA in AD transgenic mice. This is consistent with previous animal studies showing that EA improves cognitive function in animal AD models [5] and human studies showing that acupuncture enhances cognitive function in individuals with mild to moderate cognitive impairment [33–35]. Although our meta-analysis was restricted to transgenic mouse models, these findings are also consistent with rat studies reporting EA reducing tau pathology and improving cognitive functions [36, 37]. In addition, we identify distinct patterns of efficacy associated with specific acupoints. Our findings from the network meta-analysis further highlight that EA at specific single and multiple acupoints exhibits varying degrees of efficacy. This indicates the importance of targeted interventions.

As for single acupoints, EA at the GV20 acupoint consistently emerges as highly effective across multiple outcomes, including reductions in Aβ deposition, increased time spent in the target quadrant, and decreased escape latency. This result is perhaps not surprising as GV20 is the most common acupoint in both animal and human studies. These results suggest that GV20 may play a central role in modulating brain function and alleviating key symptoms of AD. Similarly, EA at ST36 is notably effective in reducing neuroinflammatory biomarkers, such as GFAP, IL-1β, and microglia activation. However, ST36’s efficacy varies across biomarkers, with more robust effects for Aβ deposition but weaker effects for other outcomes. This highlights the need for further exploration of ST36’s therapeutic potential and its variability in efficacy.

Furthermore, multi-acupoint combinations, such as GV20/GV26/GV29 and GV20/GV14, show strong synergistic effects, particularly in behavioral outcomes like memory retention and spatial learning. GV20/GV26/GV29 consistently exhibit high precision across behavioral metrics, supported by strong network connections and reliable effects. Although GV20/GV14 demonstrates slightly weaker precision, it still achieves measurable therapeutic outcomes. Interestingly, adding more acupoints does not necessarily enhance efficacy and, in some cases, leads to diminished outcomes. For example, EA at GV20 alone often outperforms combinations such as GV20/KI1 and GV20/GV24. In the time spent in the target quadrant metric, EA at GV20 alone achieves an SMD of 1.83, compared to 1.17 and 0.75 for GV20/KI1 and GV20/GV24, respectively. Similarly, for the number of platforms crossed, EA at GV20 yields an SMD of 1.37, while GV20/KI1 and GV20/GV24 show lower SMDs of 0.81 and 1.00, respectively. These findings suggest that certain acupoint combinations may interfere with or dilute the therapeutic efficacy of highly effective single acupoints like GV20. The network analysis we conducted suggests the importance of carefully selecting complementary acupoints to maximize therapeutic outcomes for AD. Further research is needed to investigate the mechanisms underlying acupoint interactions and to identify optimal combinations. By refining EA interventions, future studies can better leverage its therapeutic potential to address AD pathology and improve cognitive and behavioral outcomes.

## Supplementary information

Supplementary Information

## Data Availability

The data and code supporting the findings of this meta-analysis are available at: https://github.com/HenryYang03/Meta-Analysis.
