# Supplementary material for: Evaluating the potential of acupuncture for Alzheimer’s disease treatment: A meta-analysis and systematic review of mouse model studies
Source: Transl Psychiatry. 2026 Mar 17;16:153. doi: 10.1038/s41398-026-03923-9 (PMC13002870; doi:10.1038/s41398-026-03923-9)

**Supplementary Table 1.** Summary Table of selected publications. The table includes information on paper ID, publication year, authors, journal, title, AD model, and acupoints used for treatment.

**Supplementary Table 2.** Egger’s regression test outcomes for potential publication biases

**Supplementary Figure S1.** Venn diagrams to summarize the distribution of outcome variables

across studies. **A**: key biomarkers (Aβ, IL-1β, IBA1, and GFAP), showing how often these metrics appear alone or in combination across included papers. **B**: behavioral metrics (time spent in the target quadrant, number of platform crossings, escape latency, and discrimination index), reflecting the overlap of reported outcomes in cognitive and behavioral experiments.

**Supplementary Figure S2. A-D**: Forest plot for the effect of EA treatment on Aβ deposition among four subgroups. (SMD = (Treatment Group’s outcome - Control Group’s Outcome) / SD pooled)

**Supplementary Figure S3.** The supplementary figures provide a day-by-day breakdown of escape latency. **A-E**: escape latency forest plots from Day 1 to Day 5, respectively, with a consistent decrease in latency observed in EA-treated groups compared to controls. (SMD = (Treatment Group’s outcome – Control Group’s Outcome) / SD pooled) forest plots.

**Supplementary Figure S4.** Outlier removal results for each outcome variable.

**Supplementary Figure S5. A-D**: Forest plot for the effect of EA treatment on the number of platforms crossed among four subgroups.

**Supplementary Figure S6. A**: Leave-one-out influence analysis for time spent in the target quadrant. **B-D**: Forest plot for the effect of EA treatment on the time spent in the target quadrant among three subgroups.


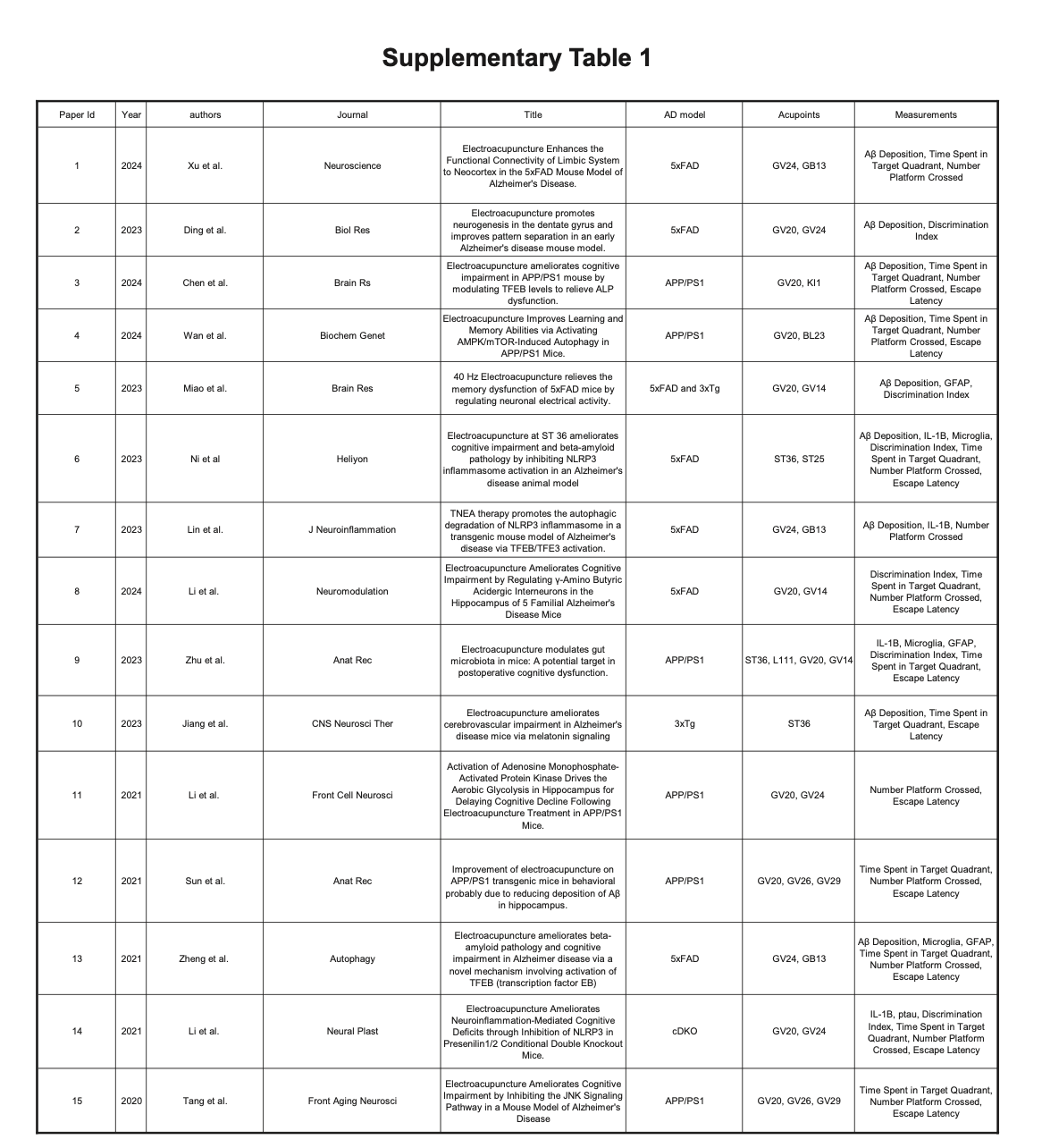


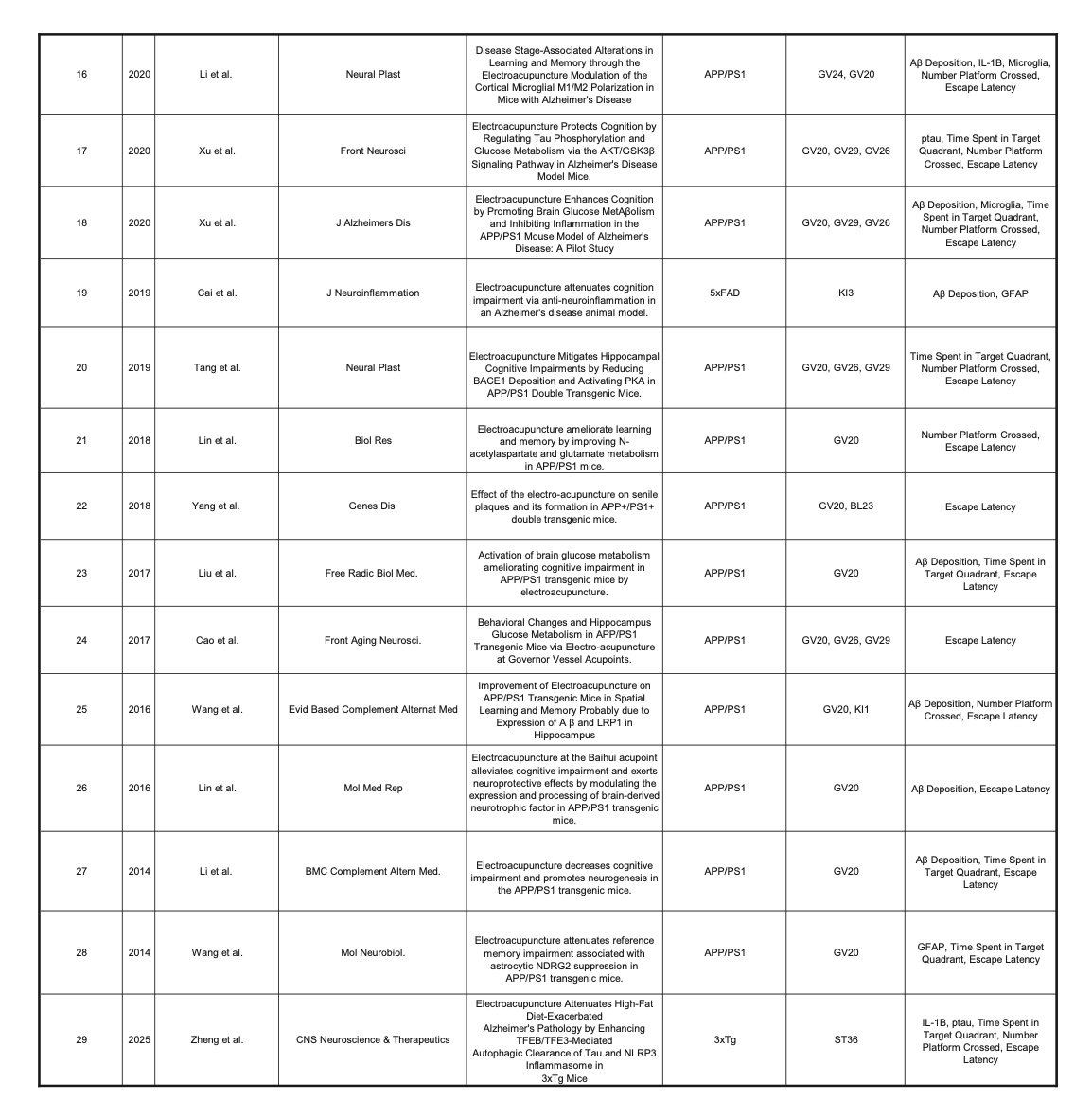


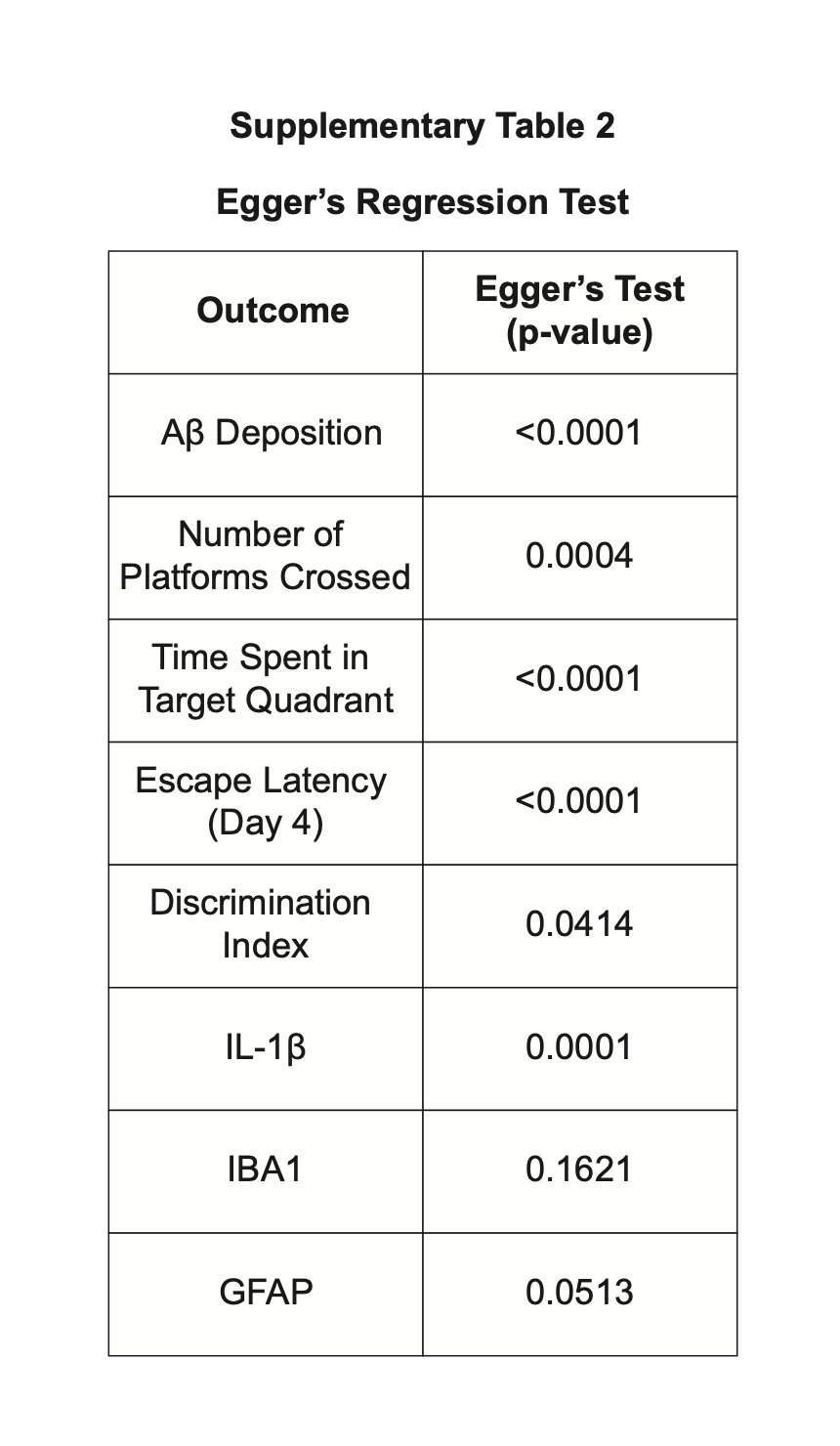


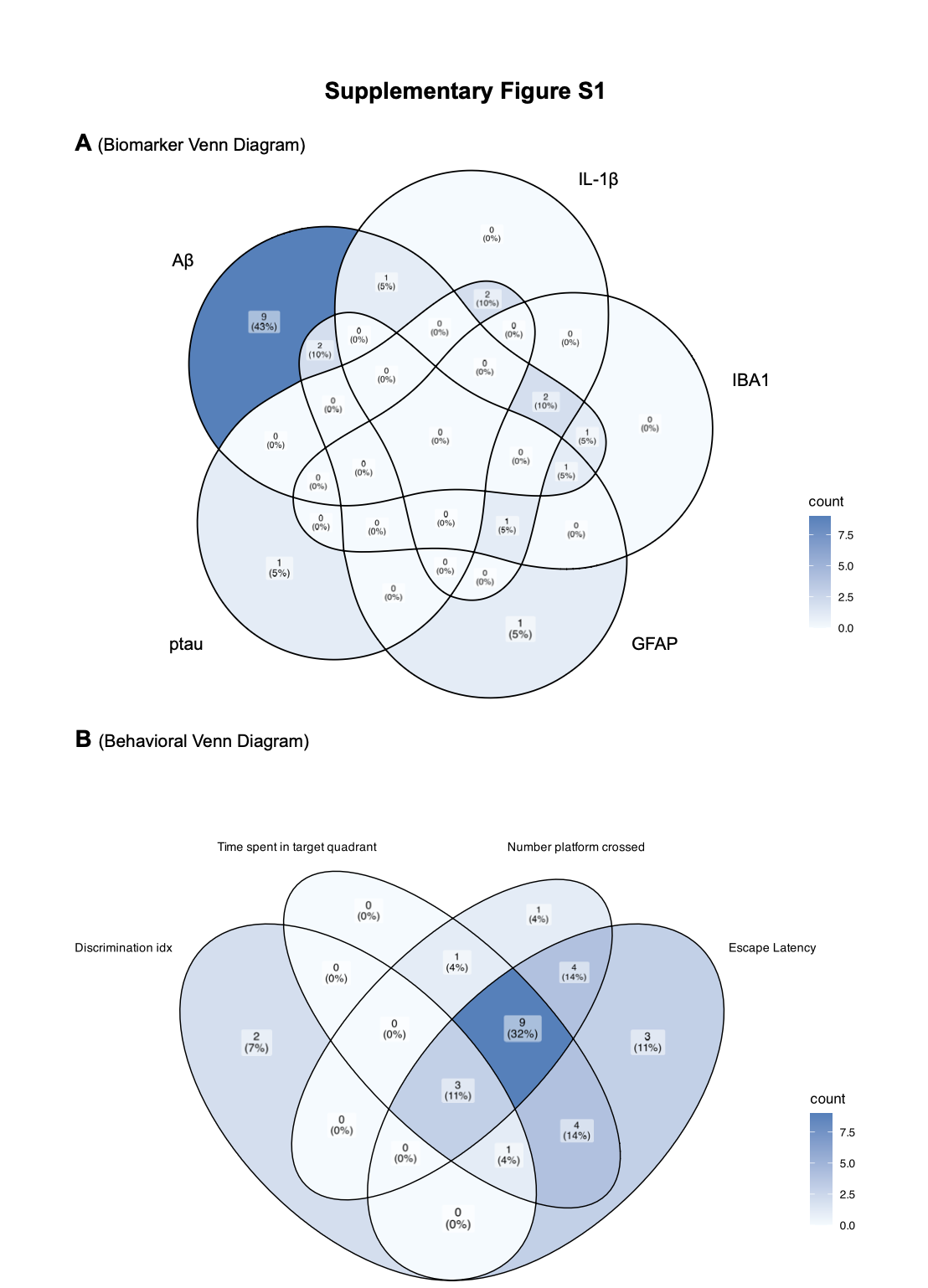


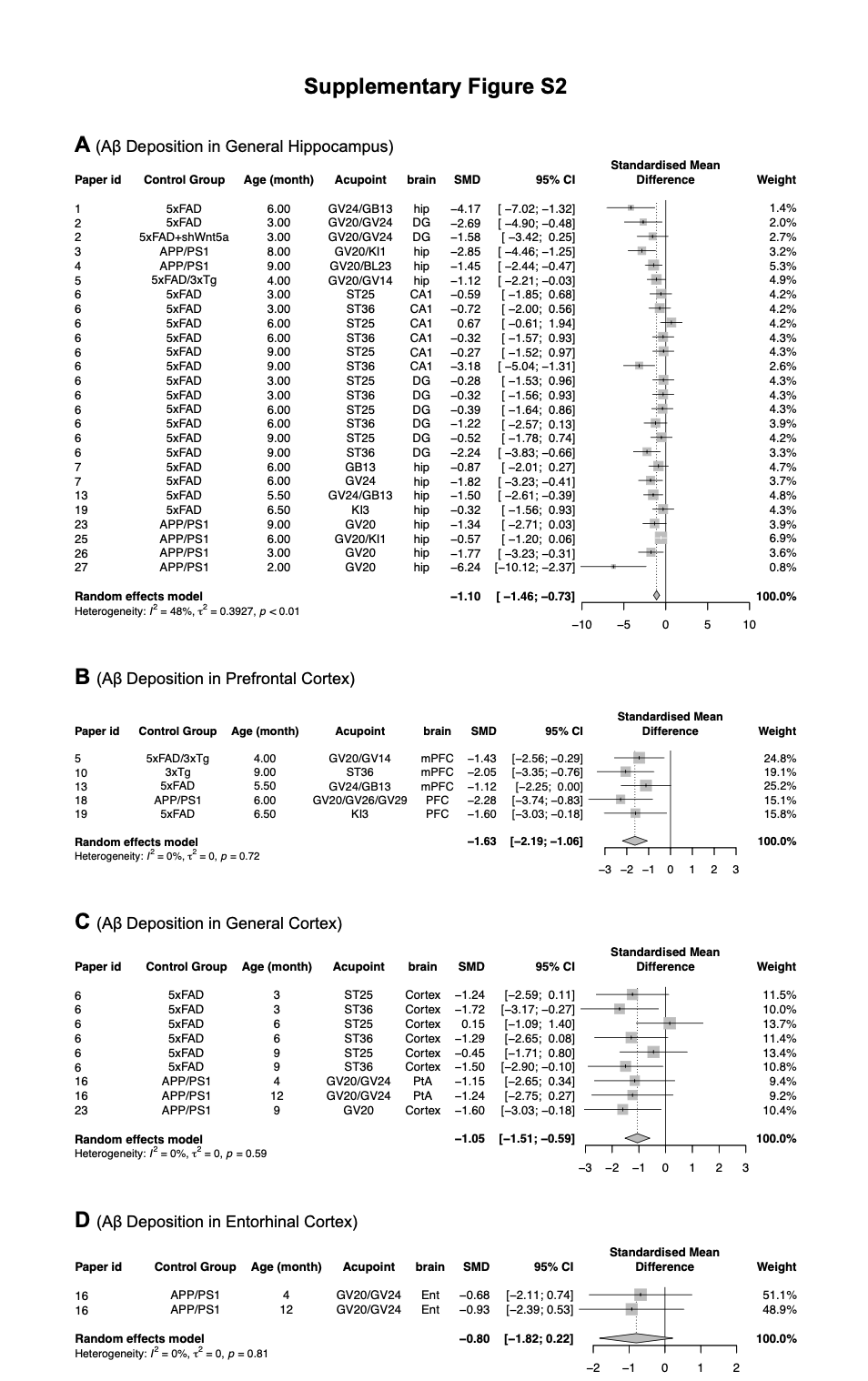


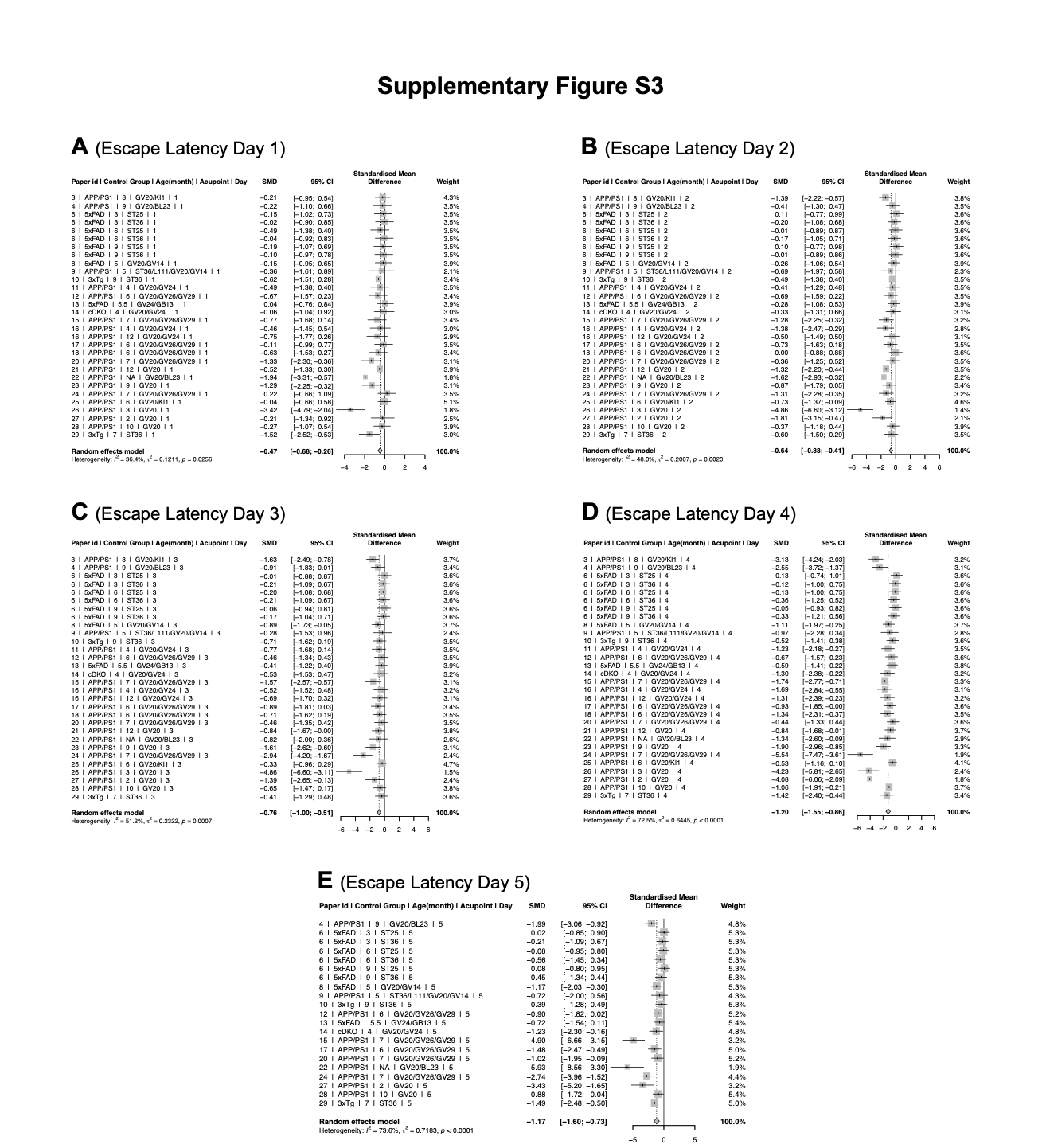


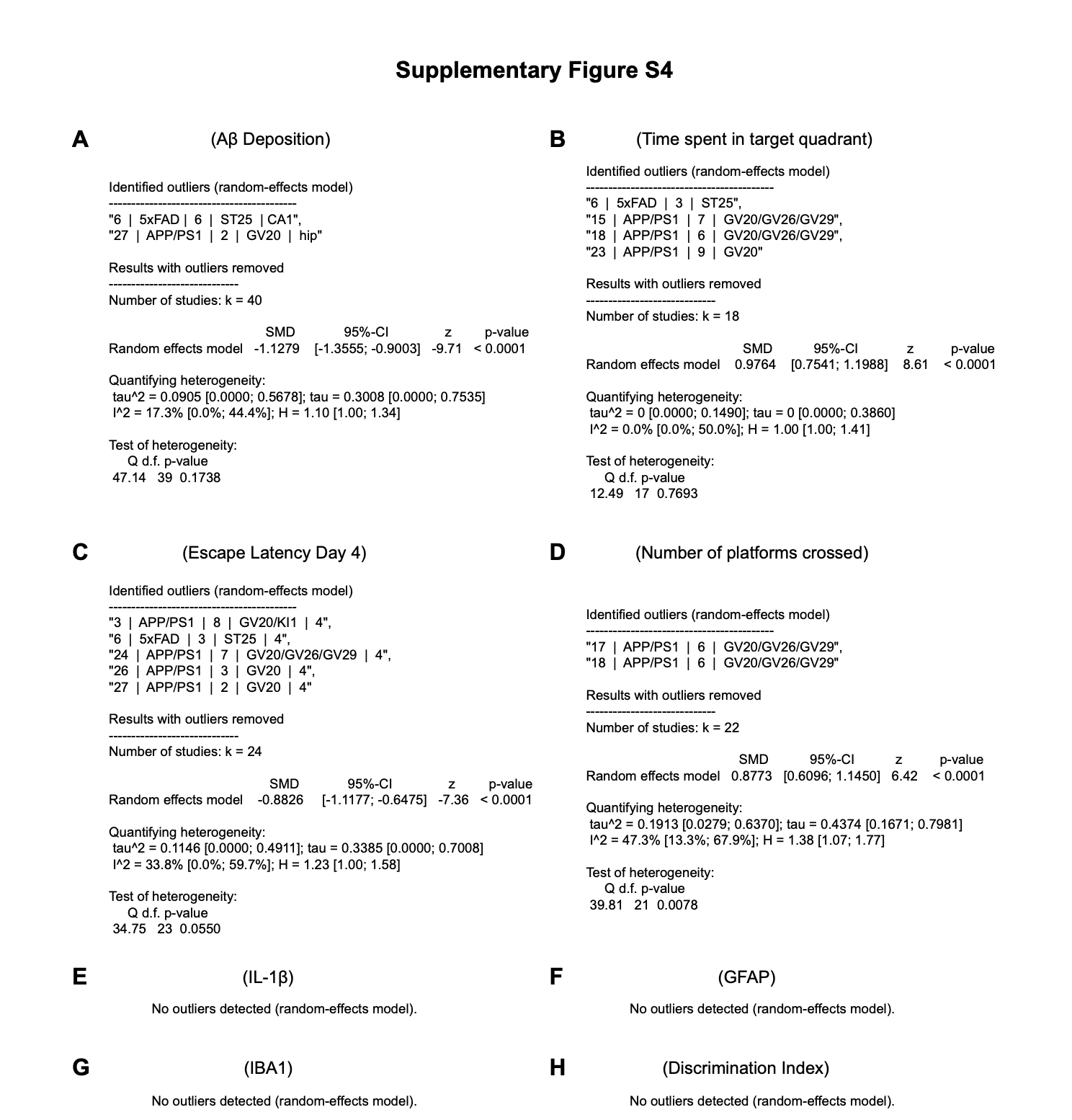


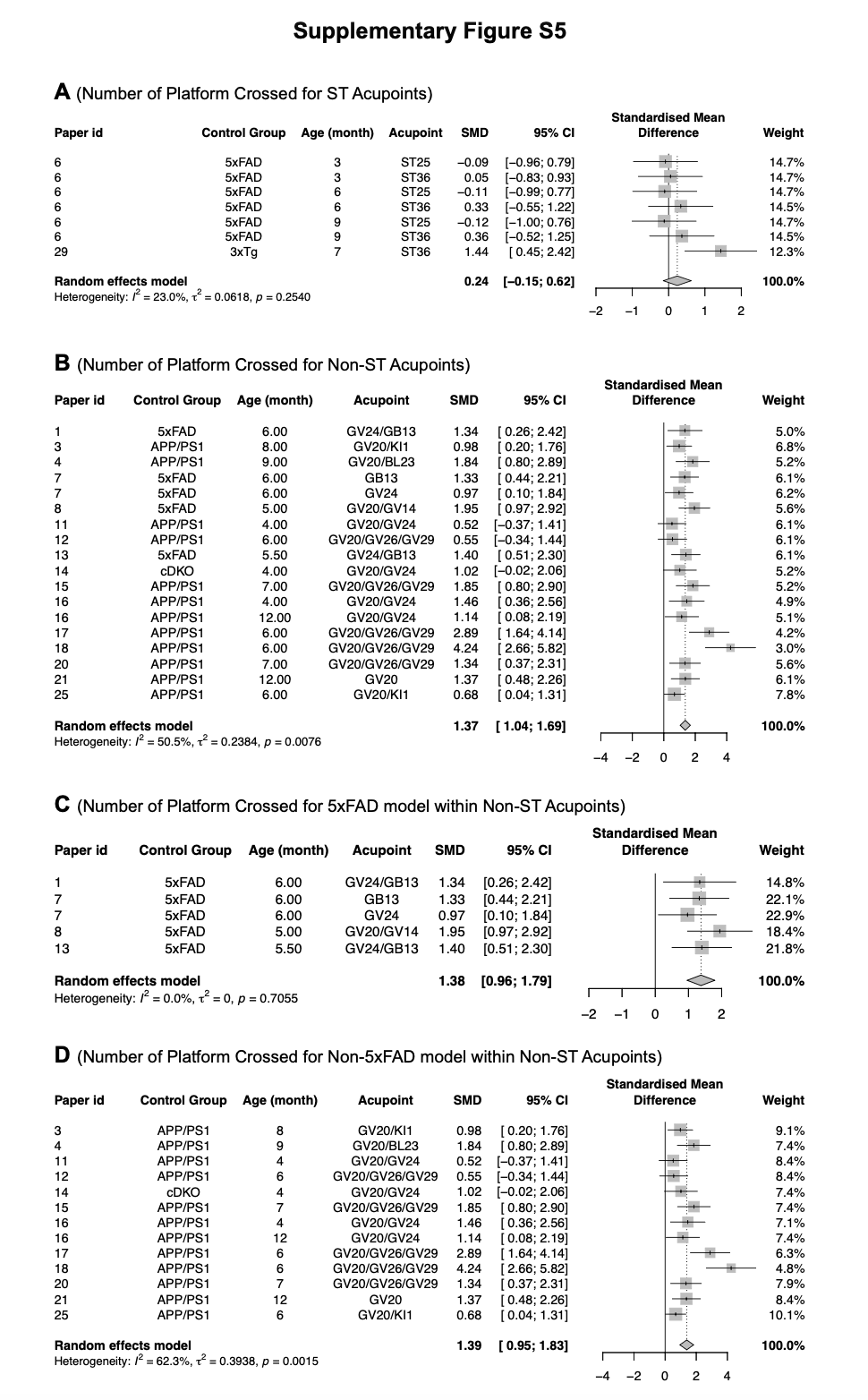


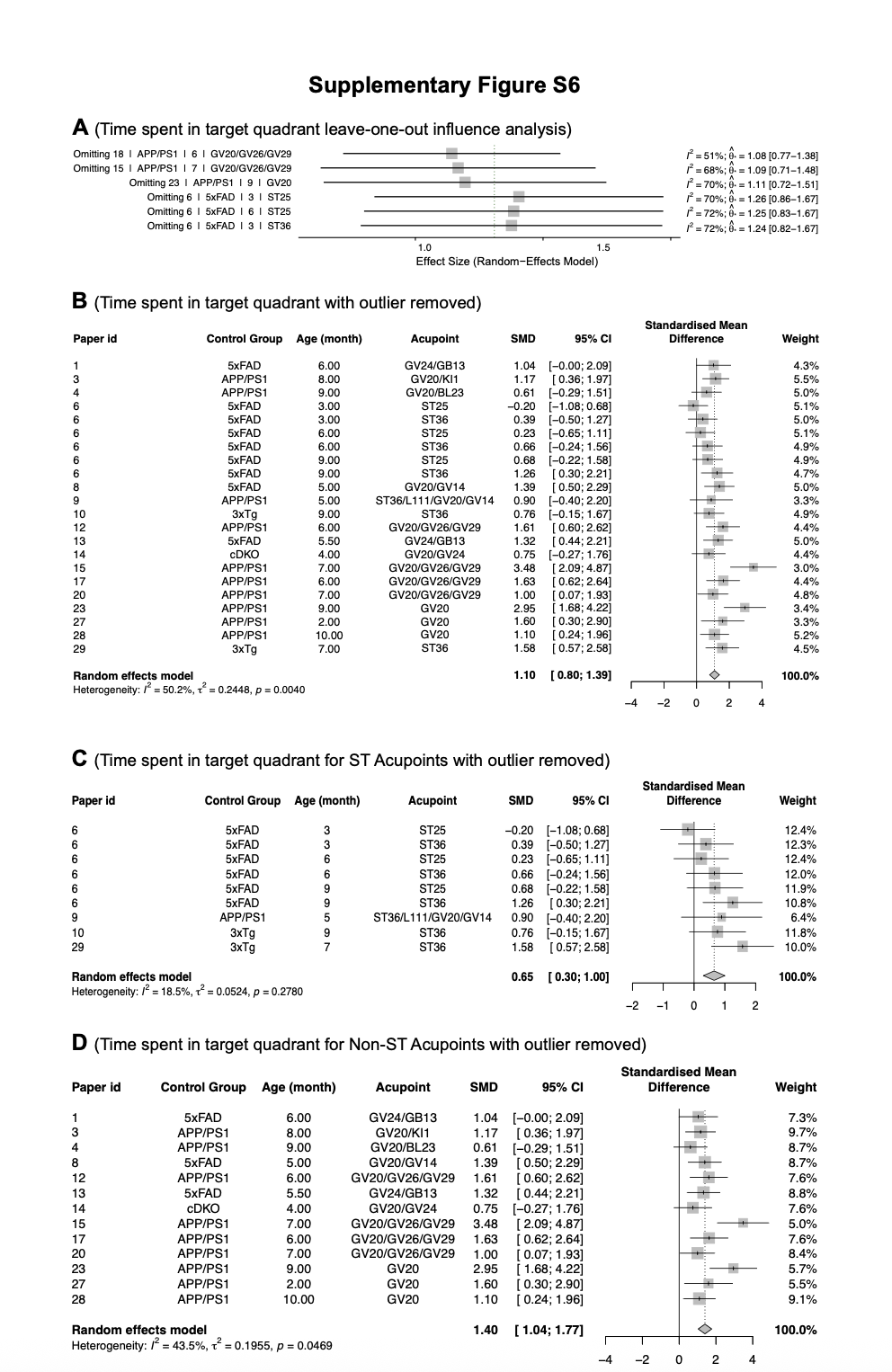

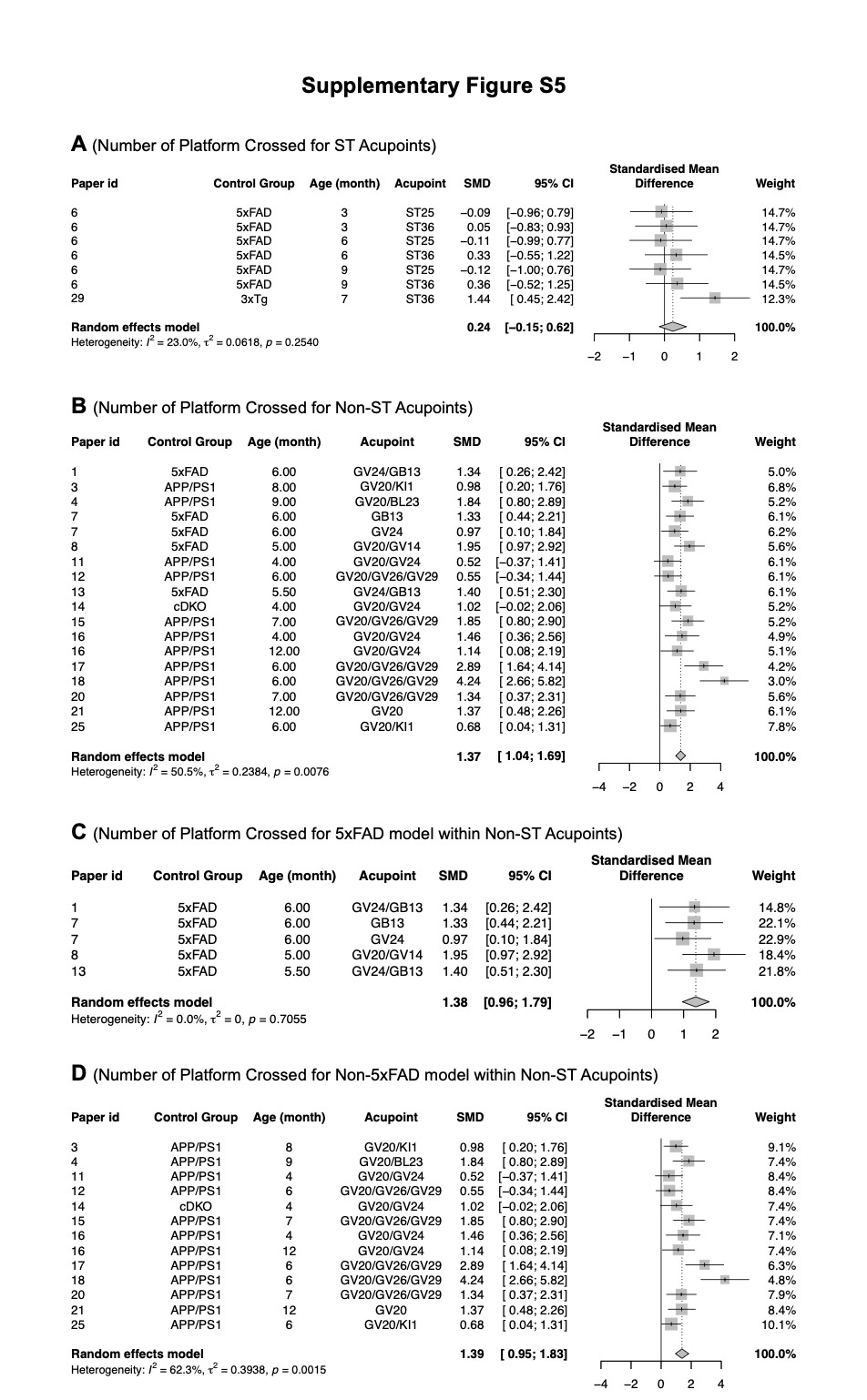

Supplement: Supplementary file 1 — Supplementary Information [file 41398_2026_3923_MOESM1_ESM.docx]
